# Genetic Variation of the Fc Gamma Receptor 3B Gene and Association with Rheumatoid Arthritis

**DOI:** 10.1371/journal.pone.0013173

**Published:** 2010-10-05

**Authors:** Rute B. Marques, Mohamed M. Thabet, Stefan J. White, Jeanine J. Houwing-Duistermaat, Aleida M. Bakker, Gert-Jan Hendriks, Alexandra Zhernakova, Tom W. Huizinga, Annette H. van der Helm-van Mil, Rene E. Toes

**Affiliations:** 1 Department of Rheumatology, Leiden University Medical Center, Leiden, The Netherlands; 2 Department of Internal Medicine, Assiut University Hospital, Assiut, Egypt; 3 Molecular Development, Murdoch Childrens Research Institute, Melbourne, Australia; 4 Department of Medical Statistics, Leiden University Medical Center, Leiden, The Netherlands; Worcester Polytechnic Institute, United States of America

## Abstract

**Background:**

Fc gamma receptors (FcγRs) play a crucial role in immunity by linking IgG antibody-mediated responses with cellular effector and regulatory functions. Genetic variants in these receptors have been previously identified as risk factors for several chronic inflammatory conditions. The present study aimed to investigate the presence of copy number variations (CNVs) in the *FCGR3B* gene and its potential association with the autoimmune disease rheumatoid arthritis (RA).

**Methodology/Principal Findings:**

CNV of the *FCGR3B* gene was studied using Multiplex Ligation Dependent Probe Amplification (MLPA) in 518 Dutch RA patients and 304 healthy controls. Surprisingly, three independent MLPA probes targeting the *FCGR3B* promoter measured different CNV frequencies, with probe#1 and #2 measuring 0 to 5 gene copies and probe#3 showing little evidence of CNV. Quantitative-PCR correlated with the copy number results from MLPA probe#2, which detected low copy number (1 copy) in 6.7% and high copy number (≥3 copies) in 9.4% of the control population. No significant difference was observed between RA patients and the healthy controls, neither in the low copy nor the high copy number groups (p-values = 0.36 and 0.71, respectively). Sequencing of the *FCGR3B* promoter region revealed an insertion/deletion (indel) that explained the disparate CNV results of MLPA probe#1. Finally, a non-significant trend was found between the novel -256A>TG indel and RA (40.7% in healthy controls versus 35.9% in RA patients; P = 0.08).

**Conclusions/Significance:**

The current study highlights the complexity and poor characterization of the *FCGR3B* gene sequence, indicating that the design and interpretation of genotyping assays based on specific probe sequences must be performed with caution. Nonetheless, we confirmed the presence of CNV and identified novel polymorphisms in the *FCGR3B* gene in the Dutch population. Although no association was found between RA and *FCGR3B* CNV, the possible protective effect of the -256A>TG indel polymorphism must be addressed in larger studies.

## Introduction

**Figure 1:**
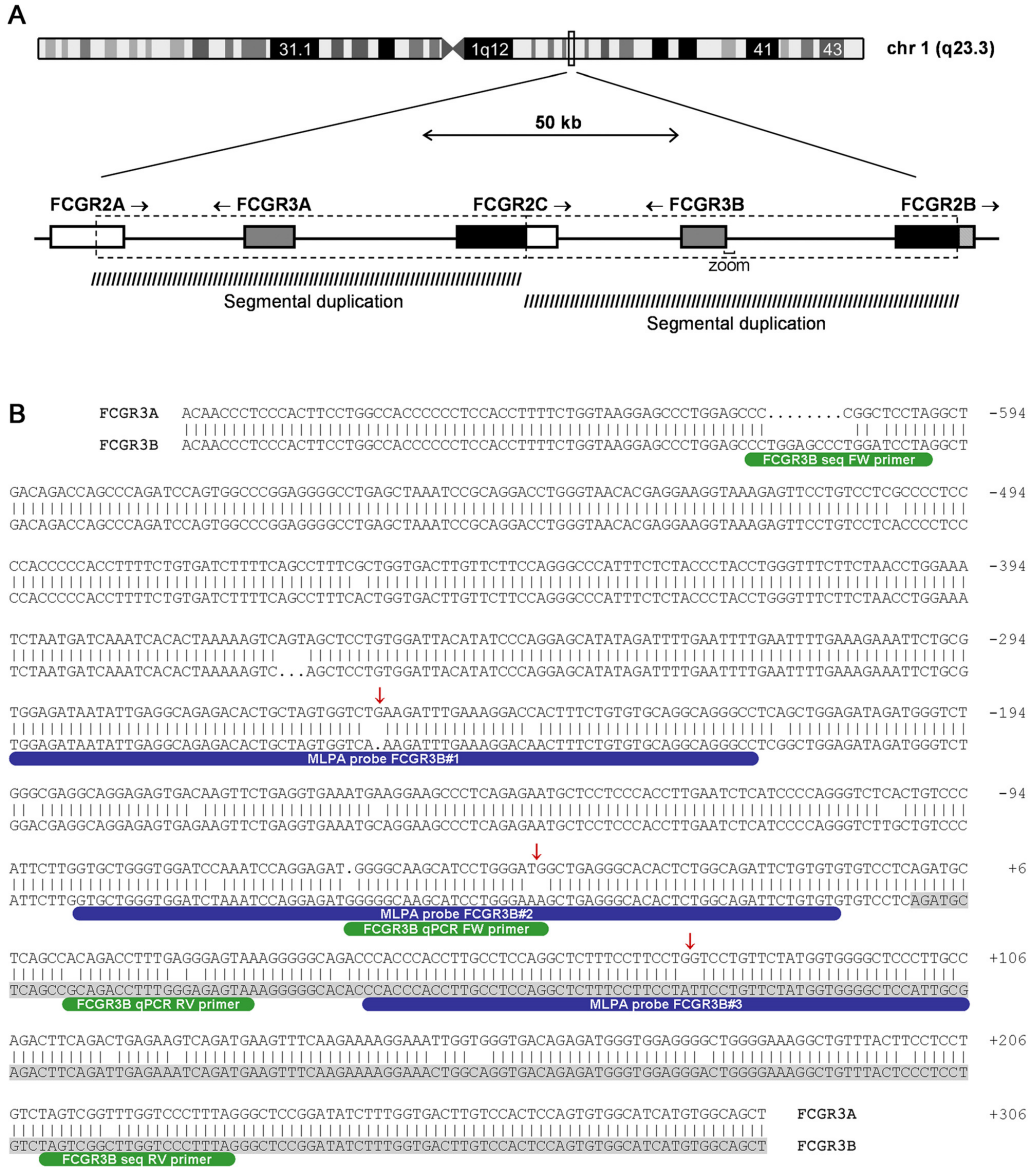
Genomic organization of the human FCGR locus in the chromosome 1q23.3. **A**. *FCGR2A*, *FCGR2B*, *FCGR2C*, *FCGR3A* and *FCGR3B* are drawn in different shades of gray, representing the regions of homology between the genes. Arrows mark the direction of transcription. MLPA probes designed to measure *FCGR3B* copy number are located in the promoter region (zoom). **B**. Zoom of the promoter sequence of *FCGR3B* aligned against the homologous *FCGR3A* gene. The first exon of *FCGR3B* is highlighted in grey, MLPA probes in blue, quantitative PCR and sequencing primers in green. Red arrows mark the ligation site of the MLPA probes, which target paralogous sequence variations between the *FCGR3A* and *FCGR3B* genes to assure specificity.

Fc receptors are proteins expressed on the surface of immune cells, whose function is to help in the recognition and elimination of invading pathogens [1]. Fc receptors bind to antibodies attached on the surface of pathogens or infected cells, triggering immune effector responses, such as phagocytosis, antibody-dependent cellular cytotoxicity, cytokine release and antigen presentation. There are Fc receptors for each immunoglobulin (Ig) class: FcαR, FcδR, FcεR, FcγR and FcµR, for IgA, IgD, IgE, IgG and IgM, respectively. IgG antibodies are the most abundant serum immunoglobulins, are predominantly involved in the secondary immune response and increased amounts can occur upon infection, chronic inflammation and autoimmune diseases. Therefore, FcγRs are thought to play a crucial role in immunity, as well as in the pathogenesis of several autoimmune diseases, including rheumatoid arthritis (RA) [2]. FcγRs vary in their cellular distribution and affinity for different IgG isotypes and can be divided in three general classes: FcγRI (isoforms FcγRIA, IB and IC), FcγRII (isoforms FcγRIIA, IIB and IIC) and FcγRIII (isoforms FcγRIIIA and IIIB). These include activatory receptors, such as FcγRI, FcγRIIA and FcγRIIIA, and the inhibitory receptor FcγRIIB [3]. Furthermore, FcγRs can be distinguished between high-affinity receptors (FcγRI) and low-affinity receptors (FcγRII and FcγRIII). These low-affinity receptors are encoded by highly homologous FCGR genes, located in a genetically complex cluster on the long arm of chromosome 1 (Figure 1) [4]. It is believed that the different *FCGR* genes in this locus are the result of multiple duplication and recombination events during evolution [5]. Additionally, this region displays extensive genetic variation, which has been associated with susceptibility to various chronic inflammatory disorders [6]–[8]. In particular, single nucleotide polymorphisms (SNPs) in *FCGR2A* (R131H), *FCGR2B* (I232T) *FCGR3A* (V158F) and *FCGR3B* (NA1/NA2), have been reported in association with systemic lupus erythematosus (SLE), rheumatoid arthritis (RA) and/or idiopathic thrombocytopenia purpura (ITP) [9]–[17].

Although less studied than SNPs, copy number variants (CNVs) are also important sources of genetic variation. A CNV is defined as a sequence of DNA >1 kb that is present in altered copy number when compared with a reference genome [18]. Several recent studies have demonstrated that some genes or groups of genes can show variation in copy number [19]–[24]. In total, copy number variable regions may cover as much as 12% of the human genome, many of which exist with relatively high frequency (>5%) in general human populations and are also present at orthologous loci in other species [18], [20], [25]. The first evidence that copy-number alterations can influence human phenotypes came from sporadic diseases, termed ‘genomic disorders’, caused by de novo structural alterations [26]. The number of genomic disorders has grown, with several dozen reported to date [27]. In addition to such sporadic diseases, inherited CNVs have been found to underlie Mendelian diseases in several families [28]–[30]. Nonetheless, CNVs have been implicated in only a few percent of the 2,000 or more Mendelian diseases so far explained at a molecular level. Little is known about the genetic basis of common, complex phenotypes, and it is premature to predict the relative proportion of complex disease explained by SNPs and CNVs. In principle, complex disease might be more susceptible to ‘soft’ forms of variation such as variation in non-coding sequences or copy number which alter gene dose without abolishing gene function. Common CNVs have been reported to be associated with several complex disease phenotypes including HIV acquisition and progression [31]–[33]. Furthermore, CNVs have been implicated in several autoimmune diseases. For example, lower CN of *FCGR3B* and complement component *C4* genes have been associated with SLE susceptibility [32]–[35]. Higher CN of *CCL3L1* has been suggested as a risk factor for developing RA [36]. Additionally, CNV of *FCGR3B* was associated with microscopic polyangiitis and Wegener's granulomatosis [35].

Given the important role of FcγRs in immunity, we are interested in whether genetic variation in these genes associates with susceptibility to rheumatoid arthritis (RA). In a previous study, we showed that *FCGR3A* 158V/V genotype but not CNV conferred risk to anti-citrullinated peptide antibodies (ACPA) positive RA [37]. In the present report, we focused on the *FCGR3B* gene.

## Materials and Methods

### Patients and healthy controls

Patients were 518 Dutch Caucasian individuals with RA who fulfilled the American College of Rheumatology (ACR) classification criteria for RA, as described elsewhere [38], [39]. Controls were 304 unrelated healthy Dutch Caucasians with no history of RA [40]. An informed written consent according to the Declaration of Helsinki was obtained for both patients and controls. The Leiden institutional review board, Commissie Medische Ethiek, approved all protocols. Anti-cyclic citrullinated peptide antibodies (ACPA) were positive in 59.6% of RA patients and was tested using Immunoscan CCPlus (CCP2 Euro-Diagnostica, Arnhem, the Netherlands).

### Multiplex ligation-dependent probe amplification

**Figure 2:**
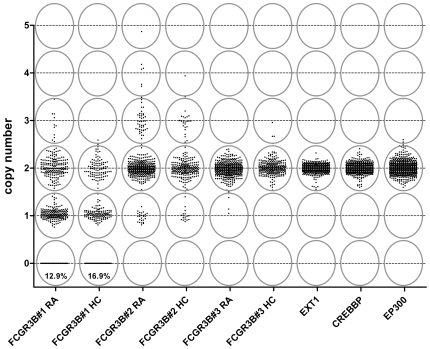
Copy number of *FCGR3B* gene in RA patients versus healthy controls. The normalized MLPA ratios measured with FCGR3B#1, FCGR3B#2 and FCGR3B#3 probe-sets are plotted for RA patients and healthy controls. Three genes *EXT1*, *CREBBP* and *EP300* were used as reference for normalization (2 copies without CNV) and are shown here for all individuals. The clusters reflecting the copy numbers are illustrated by grey ovals. FCGR3B#1 and FCGR3B#2 showed clear clusters and copy numbers could be assigned, while FCGR3B#3 showed no CNV.

Copy number variation (CNV) of the *FCGR3B* gene was assessed using Multiplex Ligation-dependent Probe Amplification (MLPA), which is a sensitive method for copy number quantification [41]. MLPA probe design and assay were performed as described by White *et al.* [42], [43]. Due to the very high homology in nucleotide sequences of FCGRs genes, the choices for appropriate MLPA probes were limited. Three probe pairs were designed for *FCGR3B*, which locate to the promoter region of the gene, based on sequence information from NCBI build 36.1 and dbSNP built 126. The MLPA probe sequences used are shown in Table 1. Three genes *CREBBP*, *EXT1* and *EP300* were used as control genes in each assay. The MLPA results were analyzed as described by White et al. [42]. The height of each probe specific peak was divided by the sum of three control peaks (from *CREBBP*, *EXT1* and *EP300*), to calculate the sample to control ratio. For each target gene, the normalized ratio was calculated by setting the median ratio across all samples within an assay at the value of 2. The normalized ratio for each individual was calculated and plotted (Figure 2). Subgroups corresponding to different gene copy numbers were defined by eye and confirmed by cluster analysis (using R statistical software version 2.5.0), and are delineated by elliptical lines.

**Table 1:** MLPA probes sequences used for CNV typing of *FCGR3B* and control genes.

Probe_Orientation	Sequence
FCGR3B#1_Forward	CGTGGAGATAATATTGAGGCAGAGACACTGCTAGTGGTCA
FCGR3B#1_Reverse	AAGATTTGAAAGGACAACTTTCTGTGTGCAGGCAGGGC
FCGR3B#2_Forward	CCGACGTACGTATCTAAATCCAGGAGATGGGGGCAAGCATCCTGGGAA
FCGR3B#2_Reverse	AGCTGAGGGCACACTCTGGCAGATTCTGTGTG
FCGR3B#3_Forward	CCCCACCTTGCCTCCAGGCTCTTTCCTTCCTA
FCGR3B#3_Reverse	TTCCTGTTCTATGGTGGGGCTCCATTGCGAGA
CREBBP_Forward	CCAGCTAGTGGAATTCAAAACACAATTGGTTCTGTTGGCACA
CREBBP_Reverse	GGGCAACAGAATGCCACTTCTTTAAGTAACCC
EP300_Forward	CCAACCTAAGCACTGTTAGTCAGATTGATCCCAGCTCCAT
EP300_Reverse	AGAAAGAGCCTATGCAGCTCTTGGACTACCCTATCA
EXT1_Forward	CCTGAAGAACATGGTGGACCAATTGGCCAATTGTGAGGACAT
EXT1_Reverse	TCTCATGAACTTCCTGGTGTCTGCTGTGACAAAATTGCCTC

### Quantitative PCR

To verify the results of the different MLPA probes for *FCGR3B* gene, a quantitative PCR (qPCR) assay was designed based on primers located within the promoter region of *FCGR3B* gene, the same regions where the MLPA probes for *FCGR3B* were located. 24 samples showing different copy numbers of *FCGR3B* in the MLPA assay were analysed by qPCR. Sequences of the primers used were, FCGRIIIB_qPCR_FW (CAA GCA TCC TGG GAA AGC T), FCGRIIIB_qPCR_RV (TAC TCT CCC AAA GGT CTG C), FOXP2_FW (TGA CAT GCC AGC TTA TCT GTT T) and FOXP2_RV (GAG AAA AGC AAT TTT CAC AGT CC). Quantitative PCR was conducted in triplicate on 25 ng genomic DNA, using iQ SYBR Green Supermix (Bio-Rad) and an iCycler Real-Time PCR Detection System (Bio-Rad). PCR mix contained per sample: 10 µl SYBR Green Supermix, 2 µl (20 pmol) each primer, 1 µl water and 5 µl DNA. QPCR cycling protocol was as follows: 3 min initial denaturation at 95°C, followed by 40 cycles at 95°C for 30 sec, 63°C for 30 sec and 72°C for 45 sec, and finalized by a melt-curve analysis (55°C to 95°C, with 0.5°C increments for 10 sec). *FOXP2* was used as control for normalization (as it is known to have no CNV, reference). Copy number calculations were performed using the delta-delta Ct method, and the average copy number was set at 2.The results from qPCR were correlated with those from the MLPA assay using GraphPad Prism software v5.

### Sequencing

The MLPA assay showed inconsistent results across the different probes used for *FCGR3B* gene. Therefore, the promoter region of the *FCGR3B* gene was sequenced to verify if any kind of genetic abnormalities, such as previously unreported SNPs or insertion/deletions (indels), were present within the regions targeted by the probes. Genomic DNA (25 ng) was amplified and sequenced using the following primers: FCGR3B_seq_FW (CCT GGA GCC CTG GAT CCT A) and FCGR3B_seq_RV (CTA AAG GGA CCA AGC CGA CTA). PCR amplification was performed using a Promega GoTaq PCR kit (according to the manufacturer's standard protocol) and an Applied Biosystems 9700 Thermal Cycler. The cycling protocol comprised an initial denaturation step at 94°C for 3 min, followed by 37 cycles at 94°C for 45 sec, 64°C for 45 sec and 72°C for 1 min, followed by a final elongation step at 72°C for 7 min. PCR products were purified using a QIAGEN PCR Purification Kit, according to QIAGEN's manual. PCR products concentration and purity was determined using a Nanodrop spectrophotometer system. Sequencing was performed in an Applied Biosystems' ABI PRISM 3730 Analyzer and data was analysed with Chromas 2 (Technelysium).

### Statistical analysis

The χ2 test with 2 degrees of freedom (Epi Info v6, CDC, Atlanta, Georgia, USA) was used to compare the relation between genotypes and CNV and RA susceptibility. R statistical software (version 2.5.0) was used to cluster intensity ratios and validate the cut-offs of the discrete copy number genotypes. P-values were considered statistically significant if <0.05.

## Results

### *FCGR3B* copy number quantification and association with rheumatoid arthritis

The copy numbers of the *FCGR3B* gene were determined by MLPA using three independent probes. MLPA probe locations, targeting paralogous sequence variations between *FCGR3A* and *FCGR3B*, are depicted in Figure 1. Peak heights were normalized against 3 reference probes (*EXT1*, *CREBBP* and *EP300*), selected from known autosomal dominant disease genes, which would not be deleted or duplicated without an obvious phenotype. The normalized MLPA ratios were plotted to visualize the distinct copy numbers clusters (Figure 2). As expected, the reference genes showed no evidence of CNV. Surprisingly, the CNV frequency considerably differed between the three *FCGR3B* probes analyzed. While FCGR3B#1 and FCGR3B#2 probes revealed clear clustering indicating gene copies ranging from 0 to 5, the third probe FCGR3B#3 showed little evidence of CNV. The frequencies of copy number variations are shown in (Table 2).

**Table 2:** Copy number of *FCGR3B* determined with three independent MLPA probes.

MLPA Probe		RA		Controls		Low/high CN vs rest	
		Count	%	Count	%	P	OR (95% CI)
**FCGR3B#1**	0 Gene Copies	54	12.9%	43	16.9%	0.104	0.77 (0.55–1.07)
	1 Gene Copy	175	41.9%	113	44.3%		
	2 Gene Copies	179	42.8%	98	38.4%		
	3 Gene Copies	10	2.4%	1	0.4%	0.047	6.23 (0.82–131)
**FCGR3B#2**	Low CN	25	6.0%	17	6.7%	0.712	0.89 (0.45–1.76)
	2 Gene Copies	344	82.3%	213	83.9%		
	High CN	49	11.7%	24	9.4%	0.359	1.27 (0.74–2.2)
**FCGR3B#3**	Low CN	3	0.7%	0	0.0%	ND	ND
	2 Gene Copies	416	99.3%	252	98.8%		
	High CN	0	0.0%	3	1.2%	ND	ND

Mantel-Haenszel p-values were calculated. (ND) p-values and OR were not determined because the number of events was too low.

FCGR3B#1 probe measured copy numbers ranging from 0 to 3 copies. One copy was the most frequent copy number, occurring in 44.3% of the controls and 41.9% of the RA patients. Furthermore, the FCGR3B#1 probe reported homozygous deletion (0 copies) in 16.9% of healthy controls versus 12.9% RA patients, and gene amplification (3 copies) in 0.4% of healthy controls versus 2.4% of RA patients (OR = 6.23, P = 0.05). In contrast, FCGR3B#2 probe showed that most individuals (>80%) contained 2 copies of *FCGR3B*. High copy number (mainly 3 copies and few individuals with 4–5 copies) was present in 9.4% of controls and 11.7% of RA patients OR = 1.27, 95%CI = 0.74–2.2, P = 0.36. Conversely, low copy number (1 copy) was present in 6.7% of the controls compared to 6.0% of RA patients OR = 0.89, 95%CI = 0.45–1.76, P = 0.71.

To investigate the reason why the three FCGR3B probes, located within 500 bp from each other, produced such different results we sequenced the promoter region of *FCGR3B* and measured the number of gene copies by quantitative PCR (qPCR).

### Sequencing of the *FCGR3B* promoter region

**Figure 3:**
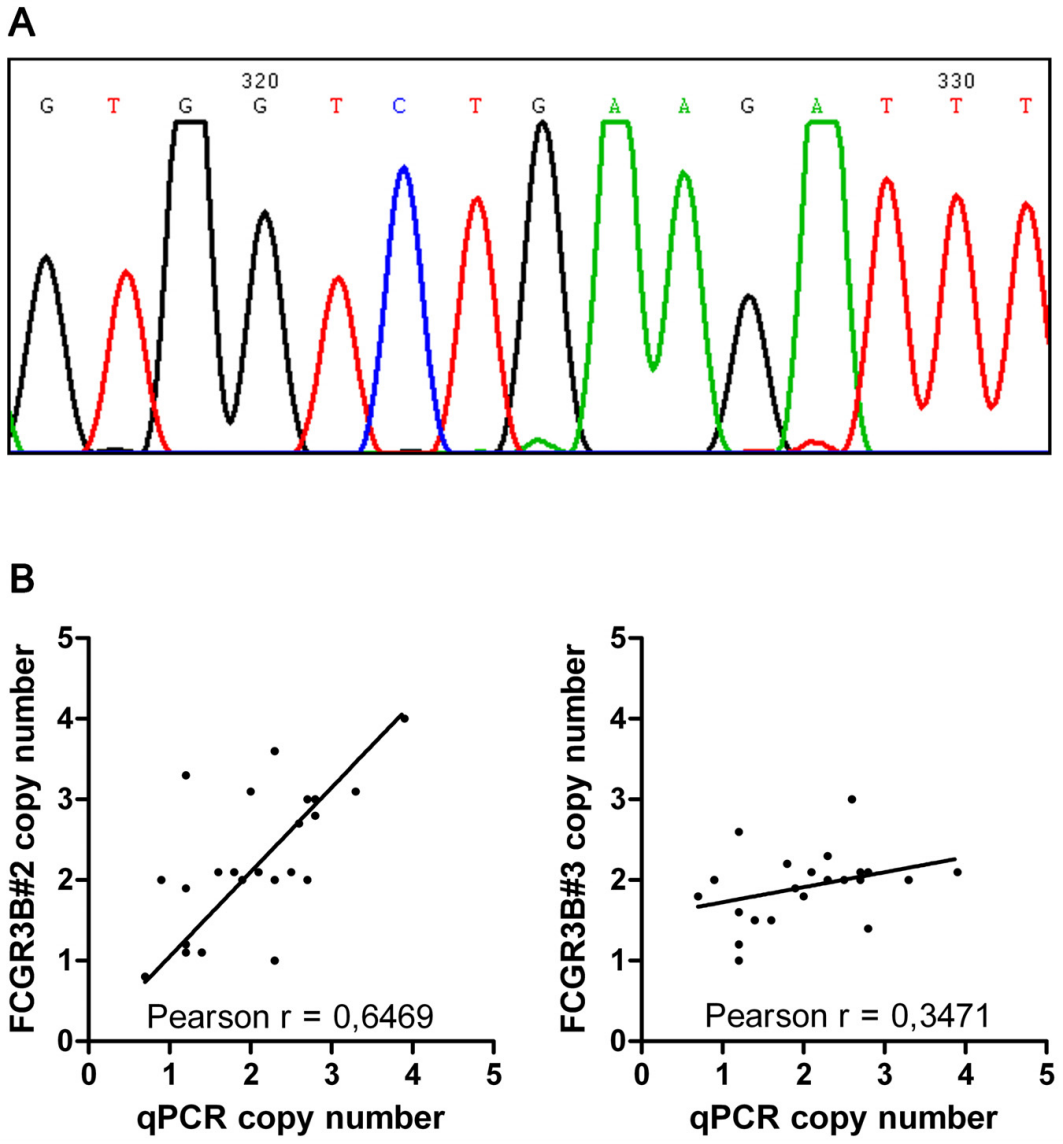
Validation of the *FCGR3B* MLPA probes. **A**. Example of the sequencing results for an individual homozygous for the novel FCGR3B-256A>TG indel at the ligation site of MLPA probe FCGR3B#1. **B**. Validation of the results of probe FCGR3B#2 (but not FCGR3B#3) by quantitative RT-PCR.

A 843 base-pair segment of the *FCGR3B* promoter encompassing the binding sites for the three MLPA probes was amplified and sequenced, to control for eventual unreported genomic variations that could affect probe ligation. Sequencing results revealed the presence of a 2 nucleotides insertion/deletion (indel) polymorphism, located exactly at the ligation site for the probe FCGR3B#1 (Figures 3A). This polymorphism consisted of the deletion of the A nucleotide at position 256 upstream of the transcription starting site, followed by TG insertion. This *FCGR3B* indel polymorphism has not been previously reported in the literature or public SNP databases, and will be referred throughout the manuscript as -256A>TG indel. The TG insertion prevents the ligation of the two FCGR3B#1 half-probes on the target sequence and subsequently, the absence of signal is misread as a lowered copy number of the *FCGR3B* gene. It is important to note that the -256A>TG indel is not a rare polymorphism, judging from the high frequency of low copy numbers measured with the FCGR3B#1 probe. Finally, these results showed that the FCGR3B#1 probe simultaneously measures CNV and the -256A>TG indel genotype of the *FCGR3B* gene, thus explaining the discordant results between that probe and the FCGR3B#2.

### *FCGR3B* copy number determination by quantitative PCR

The sequencing results explain the discrepancy between the FCGR3B#1 probe and FCGR3B#2 probe but not with FCGR3B#3. Quantitative PCR was chosen as an independent method to measure the copy number of *FCGR3B* and was performed on 24 samples (Figure 3B). The results from qPCR were correlated with those from probe FCGR3B#2, with a Pearson R of 0.65 (P = 0.0009). In contrast, the copy numbers measured with FCGR3B#3 probe did not correlate with those from qPCR Pearson R = 0.35, P = 0.1. In conclusion, FCGR3B#2 showed to be a reliable probe for CNV quantification, whereas FCGR3B#3 appeared to be not reliable for this purpose.

### Combining the data from MLPA and sequencing of *FCGR3B*

**Figure 4:**
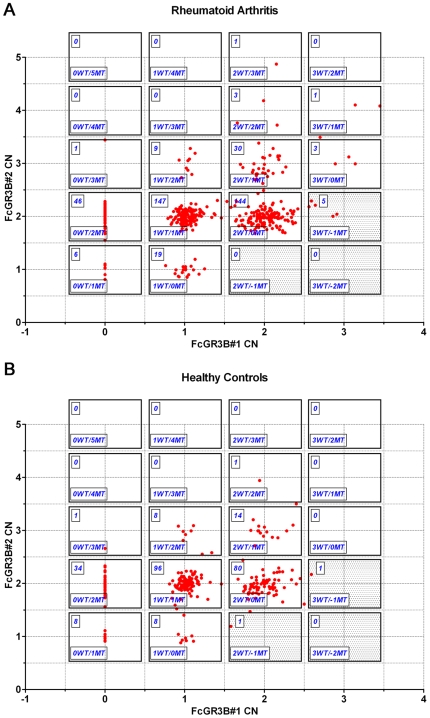
*FCGR3B* MLPA probes FCGR3B#1 versus FCGR33B#2. MLPA normalized ratios were cross-plot in RA patients (**A**) and healthy controls (**B**). Boxes delineate the clusters and the number of individuals within each cluster is shown on the upper left. WT: wild-type allele of *FCGR3B* (without the -256A>TG indel). MT: mutant-type (-256A>TG indel) allele of the *FCGR3B* gene.

The data described above indicate the presence of an additional genetic variant in the *FCGR3B* promoter. The finding of the -256A>TG indel raised the question whether this novel variant was associated with susceptibility to RA. This genetic analysis was complicated by the presence of CNV next to the -256A>TG polymorphism in the *FCGR3B* gene. Therefore, we combined the MLPA data from FCGR3B#2 probe (providing information about CNV) with the data from FCGR3B#1 probe (information about CNV and -256A>TG indel genotype). The MLPA normalized ratios were plotted in Figure 4A for the RA patients and Figure 4B for the healthy controls.

The presence of the -256A>TG indel disrupts the binding and ligation of the FCGR3B#1 MLPA probe. Therefore, this probe only measures the number of copies of the wild-type allele (without the indel). On the other hand, the FCGR3B#2 probe measures the total number of copies of the *FCGR3B* gene. The difference between the signals of FCGR3B#2 and FCGR3B#1 probes equals the number of -256A>TG alleles. For example, an individual showing 2 copies with FCGR3B#2 and 2 copies with FCGR3B#1 has 2 copies of the wild-type (WT) *FCGR3B* gene; but an individual showing 2 copies with FCGR3B#2 but only 1 copy with FCGR3B#1 has one WT allele and one -256A>TG allele. Obviously, the copy number obtained with probe FCGR3B#1 can never be higher than that determined with FCGR3B#2 probe. For this reason, 7 samples were excluded from further analysis as they measured 2–3 copies with FCGR3B#1 probe and 1–2 copies with probe FCGR3B#2, respectively, which represents a technical error rate <1% (see grey areas of the graphs on Figure 4).

Table 3 shows the frequencies of the *FCGR3B* polymorphisms, as detected by the 2 probes and stratified for the presence (mutant type: MT) or absence (wild-type: WT) of the -256A>TG indel. The WT>MT genotype was more frequent in RA patients than healthy controls (48% versus 40.8%, P = 0.1). Conversely, the WT<MT genotype was more frequent in healthy controls than RA patients (20.4% versus 15.4%). In other words, RA patients tended to have less copies of the “mutated” -256A>TG FCGR3B allele (35.9 versus 40.7%, P = 0.08), although this trend did not reach statistic significance. Stratification of the RA patients for the presence of ACPA antibodies did not change the results (data not shown).

**Table 3:** *FCGR3B* gene stratified for the -256A>TG indel polymorphism.

	RA		Controls	
	Count	(%)	Count	(%)
**WT>MT**	197	(48%)	102	(40.8%)
**WT = MT**	150	(36.6%)	97	(38.8%)
**WT<MT**	63	(15.4%)	51	(20.4%)
**MT allele**	306	(35.9%)	207	(40.7%)
**WT allele**	546	(64.1%)	302	(59.3%)
**OR (95% CI)**	0.82 (0.65–1.03)			
**Allelic P-Value**	0.08			
**Genotypic P-value**	0.12			

WT  =  Wild-type FCGR3B allele (adenine at position −256 of FCGR3B gene).

MT  =  Mutant type of the FCGR3B gene (deletion of the adenine nucleotide at position −256, followed by the insertion of thymine/guanine dinucleotide).

## Discussion

DNA segments in sizes ranging from kilobases to megabases can vary in copy number between different individuals [18], [20], [25]. These CNVs are an important source of genetic variation, which are not only responsible for population diversity but may also contribute to the inter-individual variability in disease susceptibility. A recent study of gene expression variation as a model of complex phenotype measured the fraction of gene expression ‘traits’ that could be associated with either single nucleotide polymorphisms (SNPs) or CNVs [44]. In this study, SNP genotypes and CNV measurements were associated with 83% and 18%, respectively, of those gene expression traits for which statistically significant associations were found. This may still underestimate the role of CNVs, given the higher frequency of SNPs as well as the greater completeness and accuracy with which SNPs can be queried at present [43], [45].

Curiously, there appears to be an enrichment of CNVs in genomic regions encoding immune response genes and secreted molecules [18], [46]. This finding suggests that CNVs affecting expression of immunity genes may play a functional role in infectious and autoimmune diseases, in particular. Supporting this hypothesis are previous publications describing association of CNVs in *FCGR3B* and complement component 4 (*C4A*/*C4B*) with systemic lupus erythematosus (SLE), *FCGR2C* with idiopathic thrombocytopenic purpura (ITP), defensin beta (*DEFB*) with psoriasis and Crohn disease, *CCL3L1* with HIV-AIDS infection, SLE, rheumatoid arthritis (RA) and type 1 diabetes (reviewed in [47]). On the other hand, two recent genome wide association studies (GWAS) of CNVs suggest that this type of variation is unlikely to identify novel genetic loci associating with common human diseases [48], [49]. This conclusion was based on two key findings: (i) most of the common CNVs (about 75% with MAF>10%) are well-tagged by SNPs (r2>0.8) and have already been indirectly screened in existing SNP GWAS studies; (ii) the number of confirmed disease associating loci in the CNV GWAS was much smaller than the number of loci identified in SNP GWAS, and consisted of already known associations (HLA for RA, T1D and Crohn's disease; *TSPAN8* for T2D; *IRGM* for Crohn's disease) [47]. However, rare CNVs (MAF<10%) are poorly tagged by SNPs and may have been missed in SNP-based GWAS, which encourages further CNV genotyping studies. Additionally, regions that have increased recombination, e.g. duplicons that include the *FCGR3B* gene, are unlikely to be tagged well.

In the current study we investigated the association of CNVs of the FCGRs genes with RA susceptibility. Several methods can be used to quantify CNVs, including fluorescence in situ hybridization, PCR, sequencing and genome-wide array based approaches [50]. In the present study we have chosen for multiplex ligation-dependent probe amplification (MLPA), a simple high-throughput method, which has the advantages of consuming relatively low amounts of genomic DNA and a cost-effective multiplexing potential. MLPA utilizes a ligation step to join two separate oligonucleotide half-probes after hybridization [41]. This provides increased sensitivity to distinguish highly homologous sequences, by designing the half-probes such that a difference in sequence is located under or near the ligation site [51]. However, unsuspected sequence changes at or near the ligation site may lead to erroneous interpretations, as these disturb the ligation reaction and may result in the absence of signal, which is consequently misunderstood as a deletion [52], [53]. This limitation is not restricted to the MLPA assay but to most primer/probe based assays. Thus a deletion detected with a single probe set needs to be confirmed with another assay, for example with quantitative PCR.

The FCGR locus on chromosome 1q23 originated from segmental duplication and cross-over events, which resulted in high homology between the five low affinity FCGR genes [4]. This high homology, together with the presence of multiple (uncharacterized) SNPs, makes it extremely difficult to distinguish between these genes and to design probes for genotyping assays, which may lead to erroneous interpretations [54]. During the preparation of this manuscript, three independent studies report on the *FCGR3B* gene copy number in association with the risk of developing autoimmune diseases, including RA [48], [55], [56]. The results of these studies are conflicting: while McKinney *et al.* observed a significant association of low *FCGR3B* gene copy number (CN<2) with RA, that was not the case in the studies by Mantani *et al.* and the CNV GWAS from the Wellcome Trust Consortion [48], [55], [56]. These conflicting reports further strengthen the notion that, due to a high degree of homology, CNV in this *FCGR* locus is difficult to measure and results are sometimes difficult to interpret. It could be argued that, having a smaller sample size, our present study (518 RA patients and 304 healthy controls) and that of Mantani *et al*. (158 cases and 409 controls) had limited power to detect a significant association, compared to the study of McKinney *et al*. (in total, 1661 cases and 1374 controls from New Zeeland, the Netherlands and the UK) [55], [56]. Nonetheless, our present study had a power of 90% to detect an OR = 2.01 and a power of 70% for OR = 1.67, which are the effect sizes reported by McKinney *et al*. in the “Dutch RA” and “Combined RA” populations, respectively [55]. Also, it should be noticed that the OR we detected (0.89, confidence interval 0.45–1.76) is not in the same direction as the effect reported by McKinney *et al*. Finally, the study from the Welcome Trust Case Control Consortium was the largest CNV study reported to date, including 2000 RA cases and 3000 controls [48]. Therefore, we believe that the lack of association cannot merely be explained by an insufficient statistical power of these studies. Nevertheless, it remains difficult to explain why two Dutch cohorts (ours and that of McKinney *et al*.) yield different results. We feel that the most likely explanation is found in the different methods and probes used in each study to target this complex region. The quantitative real-time PCR assay used by Mantani *et al*. to measure *FCGR3B* CNV targeted the same sequence as our FCGR3B#2 probe and yielded similar results [56]. McKinney *et al*. also used a quantitative PCR based technique, but the assay used may not be specific for *FCGR3B*, due to the presence of *FCGR3A* SNPs (rs57263253 and rs60966737) in one of the TaqMan primers [55]. Since the frequency of these potential SNPs is unknown, it is difficult to ascertain the possible effect they could have on the CNV measurement. Finally, the GWAS case control study from the Wellcome Trust Consortion used a comparative genomic hybridization (CGH) microarray approach, but the probe used in this region was not specific for the *FCGR3B* and could not distinguish from the *FCGR3A* gene [48]. Thus, it is possible that the disparate results between the different studies are due to technical issues that hamper accurate CNV measurement in this locus. Additionally, we identified a novel insertion/deletion polymorphism (-256A>TG) in the promoter region of the *FCGR3B* gene. The finding of FCGR CNVs and novel polymorphisms adds an extra level of complexity to this already intricate region. To illustrate this problem we describe here the example of our three FCGR3B probes, which all showed different copy number results in the MLPA assay. Using the human reference FCGR sequences (NCBI Build 36.1) and SNP information from dbSNP built 126, these probes were designed to be specific for *FCGR3B* gene, avoiding SNPs in *FCGR3A* that would make it “look” like *FCGR3B* and, obviously, in the absence of SNPs at the probe ligation site. However, failure of FCGR3B#1 probe in 15% of the individuals, which seemed too high a frequency for deletion, prompted us to sequence the promoter region of *FCGR3B* gene in 24 individuals. This lead to the discovery of the -256A>TG indel, a novel polymorphisms at the ligation site of the FCGR3B#1 probe. The data from FCGR3B#3 probe could not be explained by sequencing, as this probe lacked unreported SNPs or indels. Therefore, real time PCR was used quantify *FCGR3B* copy number in 24 individuals, which validated the data from FCGR3B#2 probe and excluded the results from FCGR3B#3. The strength of our study resides in the combination of these three techniques: MLPA, real-time PCR and sequencing, reaching a dept that cannot be achieved by large-scale GWAS studies.

A problem of another kind for genetic studies in copy number variable regions is caused by the presence of CNVs resulting in low SNP call rates, so that validated SNPs tend to occur with low densities in regions harboring CNVs. This is due to the fact that deletions and amplifications can lead to a skewing in the pattern of marker genotypes [20], [37]. The subsequent Hardy-Weinberg disequilibrium limits the ability of the genome-wide SNP association studies to detect disease associated SNPs in regions with CNV [45]. Additionally the presence of CNVs may blur the association of the studied SNPs with disease susceptibility [37]. Accurate quantification of counts of CNV repeats, which can be thought of as allele sizes, is not yet routinely possible; most technologies are able to quantify only the total phenotype or the sum of all alleles detected. The accurate assignment of the copy number (integer count) in an individual will present new challenges to assays, and proposals to use quantitative SNP genotypes to infer CNVs will require more-refined calling algorithms [20], [57].

In conclusion, CNV of the *FCGR3B* gene was detected in the Dutch population but did not associate with the risk of developing RA. However, a trend of association was also found for a newly identified polymorphism in the promoter region of this *FCGR3B* gene, the -256A>TG indel. Larger studies will be needed to convincingly validate these findings. Finally, the conflicting results found for the different *FCGR3B* probes illustrate the complexity of this very homologous region and the urgent need for a thorough characterization of this locus. Only so will future genetic studies of these crucial immunity genes yield consistent and reliable results.
